# What methods are used to examine representation of mental ill-health on social media? A systematic review

**DOI:** 10.1186/s40359-024-01603-1

**Published:** 2024-02-29

**Authors:** Lucy Tudehope, Neil Harris, Lieke Vorage, Ernesta Sofija

**Affiliations:** School of Medicine and Dentistry, Griffith University, Gold Coast Campus, 1 Parklands Drive, 4222 Southport, Gold Coast, QLD Australia

**Keywords:** Mental health, Stigma, Public health, Content analysis, Research methods

## Abstract

There has been an increasing number of papers which explore the representation of mental health on social media using various social media platforms and methodologies. It is timely to review methodologies employed in this growing body of research in order to understand their strengths and weaknesses. This systematic literature review provides a comprehensive overview and evaluation of the methods used to investigate the representation of mental ill-health on social media, shedding light on the current state of this field. Seven databases were searched with keywords related to social media, mental health, and aspects of representation (e.g., trivialisation or stigma). Of the 36 studies which met inclusion criteria, the most frequently selected social media platforms for data collection were Twitter (*n* = 22, 61.1%), Sina Weibo (*n* = 5, 13.9%) and YouTube (*n* = 4, 11.1%). The vast majority of studies analysed social media data using manual content analysis (*n* = 24, 66.7%), with limited studies employing more contemporary data analysis techniques, such as machine learning (*n* = 5, 13.9%). Few studies analysed visual data (*n* = 7, 19.4%). To enable a more complete understanding of mental ill-health representation on social media, further research is needed focussing on popular and influential image and video-based platforms, moving beyond text-based data like Twitter. Future research in this field should also employ a combination of both manual and computer-assisted approaches for analysis.

**Supplementary Information:**

The online version contains supplementary material available at 10.1186/s40359-024-01603-1.

## Introduction

In the last few decades, and particularly in the wake of the COVID-19 pandemic, the threat mental illness poses to public health has been increasingly recognised. The World Health Organization defines mental health as “a state of mental well-being that enables people to cope with the stresses of life, realize their abilities, learn well and work well, and contribute to their community” (World Health Organization, 2022, p. 8). However, this review is focused on mental-ill health, an umbrella term to refer to an absence of this state of well-being either through mental illness/disorder or mental health problems [1, 2]. A global burden of disease study to quantify the impact of mental and addictive disorders estimated that 16% of the world’s population were affected by some form of mental or addictive disorder in 2019, and suggest these conditions contribute to 7% of total disease burden as measured by disability adjusted life years (DALYs) [3]. Although the age-adjusted rates of DALYs and mortality for all disease causes have steadily declined in the last 15 years by 30.4% and 16.3% respectively, these rates have only increased for mental disorders by 4.3% and 12% respectively [3].

Despite the benefits and effectiveness of modern medicine, therapies and community support programs for those with mental health conditions, engagement with mental health support is often very poor [4]. Even for individuals who do eventually seek mental health care, the delay between symptom onset and treatment averages more than a decade [5]. The consequences of such delays in help-seeking can include adverse pathways to care [6], worse mental health outcomes [7], drug and alcohol abuse [8] and suicide [9]. While there are many potential barriers to the help-seeking process, significant previous research has demonstrated that attitudes towards mental illness, in particular stigma, are key factors preventing individuals from translating a need for help into action [9–11]. Stigma is a term often used in a broad sense to refer to discriminatory and negative beliefs attributed to a person or group of people [12]. However, in order to design evidence-based and effective stigma reduction interventions, a nuanced understanding of current societal views and attitudes towards mental ill-health is first necessary.

Historically, many studies investigating public stigma towards mental illness have focussed on traditional media (e.g., print or television news media), but more recently the wealth of information provided by social media has been recognised. Researchers are now harnessing social media as a powerful tool for public health research, for example in the fields of epidemiology and disease surveillance [13, 14], chronic disease management and prevention [15], health communication [16] and as an effective platform for intervention strategies [17].

Social media allows individuals to share user-generated or curated content and to interact with others [18]. It has become a central means to share their experiences and express their thoughts, opinions, and feelings towards issues. Access to such information and opinion has significant potential to influence the attitudes and health behaviours of social media users [19]. It can perpetuate negative stereotypes and increase stigma, but it can also provide a platform for discussion and sharing of personal experiences potentially helping to reduce stigma and in turn, facilitate help seeking behaviour. It must also be noted that persons living with mental illness are known to have higher rates of social media use in comparison to the general population, and are therefore at high risk of exposure to potentially negative or misrepresenting mental health content [20]. As such, social media presents a valuable research tool for investigating the attitudes of society toward mental ill-health.

Much of the previous research surrounding mental health and social media focuses on the effects of extensive social media use on psychological health and wellbeing [21] and utilizing machine learning to detect and predict the mental health status of users [22]. However, there has been a recent surge in studies using social media data to reveal attitudes and perceptions towards mental-ill health more broadly and towards specific mental health conditions. Despite the growing interest in this field and its importance to public mental health, no attempts have been made to systematically review these studies. The current state of research is heterogenous with various research designs, data collection and data analysis techniques employed to analyse social media data. A methodological review is needed to provide researchers and health professionals with an overview of the current state of the literature, demonstrate the utility of various methods and provide direction for future research.

Therefore, the aim of this systematic literature review is to provide a comprehensive overview and evaluation of the current research methods used to investigate the representation of mental ill-health on social media. The review critically appraises the quality of these studies, summarises their methodological approaches, and identifies priorities and future opportunities for research and study design.

## Methods

### Search strategy and screening procedure

Seven databases were systematically searched on September 27, 2022, including Ovid MEDLINE (via Ovid), PsycINFO (via Ovid), CINAHL (via EBSCO), SCOPUS and the ProQuest Public Health, Psychology and Computer Science Databases. Searches were filtered to present only peer-reviewed journal articles and studies published in English, and terms were applied to the title and abstract fields for each database where possible. Search terms related to [1] social media (e.g., “social platform”, “online social network*”, “user-generated”), [2] mental health (e.g., “depress*”, “anxiety”, “schizo*”) and [3] either relevant method (e.g., “(content or discourse or thematic) adj3 analy*) or terms to reflect representation (e.g., “represent*”, “attitude*”, “stigma*”). The full search strategy employed for each database can be found in Additional File 1.

The abstract and citation information for 9,576 records were downloaded and imported into Covidence systematic review software (Version 2), a web-based software specifically designed to facilitate screening, extraction, and quality appraisal. Once imported, duplicate records were automatically identified and removed by Covidence. Each stage of the screening process was carried out by two authors (LT and LV), independently. The title and abstract of 5,373 articles were screened to determine eligibility. If the two reviewers marked a different decision in Covidence, the articles were discussed and reviewers came to a consensus, and if a decision could not be made a third reviewer was consulted (ES or NH). Articles included at the title/abstract level (*n* = 136) were then screened in full text to determine relevance. Reviewers recorded the reason for exclusion. The reference list for each eligible article was then screened for any relevant publications.

**Fig. 1:**
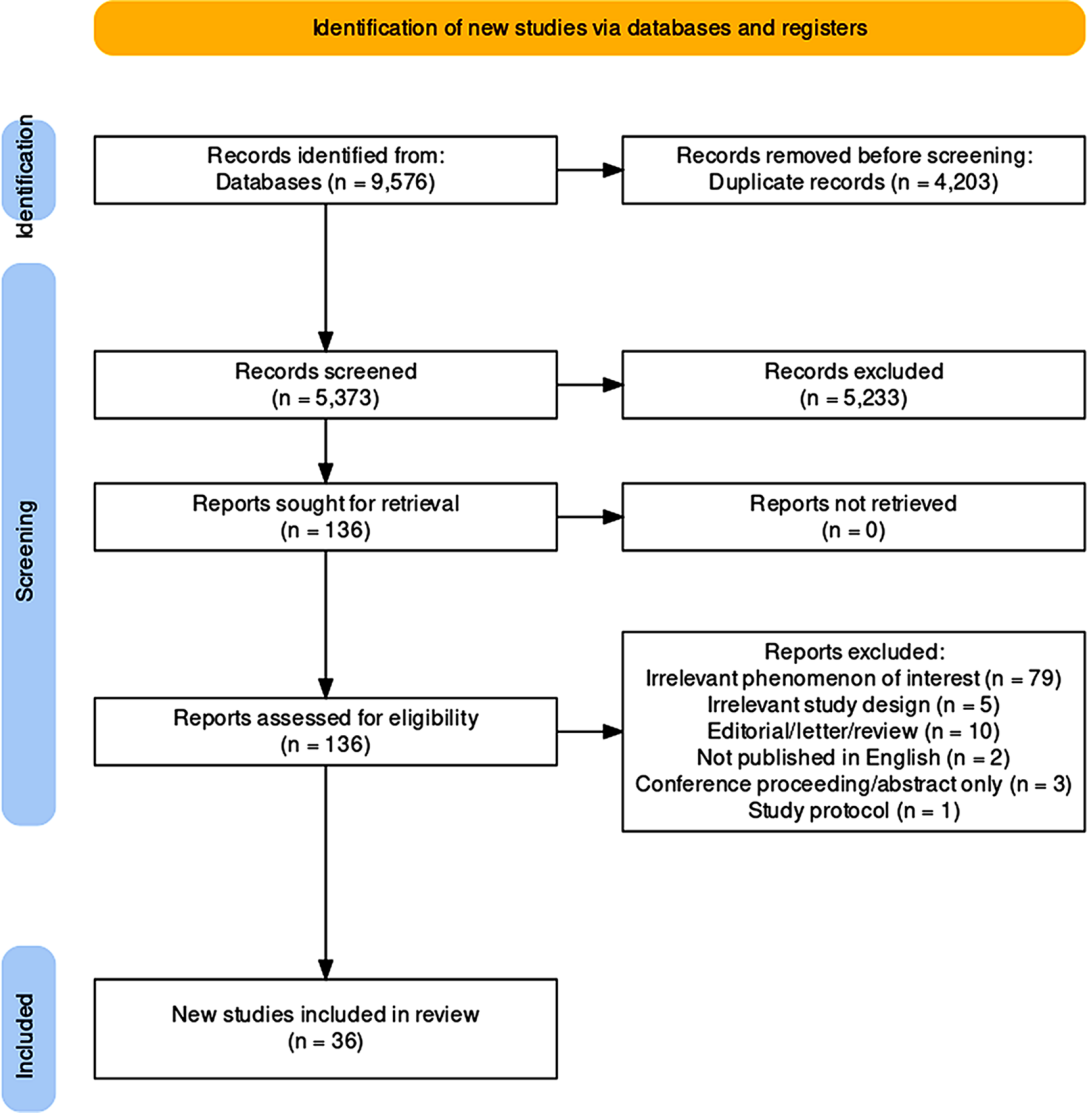
PRISMA flow diagram of identification, screening, and inclusion procedure

This systematic review is registered with the International Prospective Register of Systematic Reviews (PROSPERO, ID: CRD42022361731). The review is reported in accordance with the Preferred Reporting Items for Systematic reviews and Meta-Analyses (PRISMA) guidelines 2020 [23]. Figure 1 presents a PRISMA flowchart detailing the systematic review procedure.

### Eligibility criteria

Peer-reviewed journal articles were considered for inclusion if authors conducted an analysis of user-generated social media content regarding mental ill-health and its representation. To be considered for inclusion, social media content must be posted by individual users, as opposed to content posted on behalf of a group or organisation e.g., news media or a non-government organisation. All social media platforms except for those considered discussion forum websites such as Reddit and Quora were included. These were excluded from the review because they are considered distinct forms of social media in which content is arranged and centred on subject matter in contrast to traditional social networking sites which focus on people and their profiles. As a result, the networking dynamics are distinctly different from traditional social media platforms and bring together individuals with specific shared interests and may therefore be less appropriate for analysis of wider public perceptions and representations of mental ill-health. As per the Diagnostic and Statistical Manual of Mental Disorders, Fifth Edition (DSM-V), the scope of the systemic review was narrowed to include social media content regarding any condition classified under ‘schizophrenia spectrum and other psychotic disorders’, ‘bipolar and related disorders’, ‘depressive disorders’, ‘anxiety disorders’ and ‘obsessive-compulsive and related disorders.’ Studies must evaluate content regarding mental health more broadly or focus on a specific mental health condition as listed under these DSM-V classifications. It is beyond the scope of this review to include studies which focus on mental health in a positive sense i.e., wellbeing, happiness, and positive functioning.

In terms of study design, articles were included if they analysed the content of social media posts and/or comment responses, whether this be text, photo and/or video-based content. Data analysis methods may include but are not limited to content, discourse, thematic or linguistic analysis, and may also include studies which utilised machine learning to facilitate the analysis process. Conference proceedings, articles without accessible full-text or published in a language other than English were also excluded.

### Data extraction and synthesis

The data extraction template was developed using a sample of 5 studies. It was then piloted using an additional 5 studies and further refined. Extraction was completed through Covidence by one reviewer (LT) and subsequently checked by a second reviewer (LV). Any issues or questions were discussed and agreed upon by the two reviewers, and a third reviewer (ES or NH) was consulted if a decision could not be made. Extracted data included bibliographic information as well as methodological details, including: (1) aim/objective (2) social media platform and language (3) mental health condition/s (4) comparison to physical condition (yes/no) (5) hashtags/keywords used for search (6) data range and timeframe of collected data (7) number of posts analysed (8) type of data analysis (9) coding framework and development process and (10) coding protocol. The extracted results are presented in a narrative synthesis due to the heterogeneity of the included studies and because this review focuses on the methods of included studies.

### Article appraisal

Critical appraisal of the included studies was conducted based on the Critical Appraisal Skills Programme (CASP) guidelines for qualitative research (Critical Appraisal Skills Programme, 2022). This tool contains a checklist of 10 items which assist in the assessment of the appropriateness of the qualitative research design, consideration of ethical issues, the rigour of data collection, analysis and presentation of results and value of the research. Each item was answered with ‘Yes’, ‘Can’t tell’ or ‘No’. Two reviewers (LT and LV) independently applied the CASP checklist for each of the extracted studies. Any disagreements were discussed and resolved between the two reviewers of if this was not possible a third independent review (ES or NH) assisted.

The included studies primarily involved the analysis of text-based data derived from social media. When considering the range of critical appraisal tools which could be employed in this systematic review, the CASP tool was selected by the authors because it included items most applicable to this type of analysis, as opposed to qualitative studies involving interview or focus group-based data collection. The authors decided to exclude item 4, “Was the recruitment strategy appropriate to the aims of the research?” and item 6, “Has the relationship between researcher and participants been adequately considered?”, as there was no recruitment of active participants in the included studies. Researcher bias was instead considered when answering ‘Was the data analysis sufficiently rigorous?” by identifying whether authors demonstrated consistency in coding and factored in potential biases. The identification and selection of posts for analysis was considered in the question regarding data collection (item 5).

It must be noted that some of the studies selected for inclusion in the review analyse text-based data in a quantitative manner or conduct additional quantitative analysis of social media reach metrics. These studies were still appraised using the CASP tool, however questions such as “Is a qualitative methodology appropriate?” and “Was the data analysis sufficiently rigorous?” were modified or expanded to include consideration of any quantitative analysis elements. This was deemed more appropriate than employing a mixed-methods appraisal tool, which included items inappropriate or irrelevant to the included studies.

## Results

**Fig. 2:**
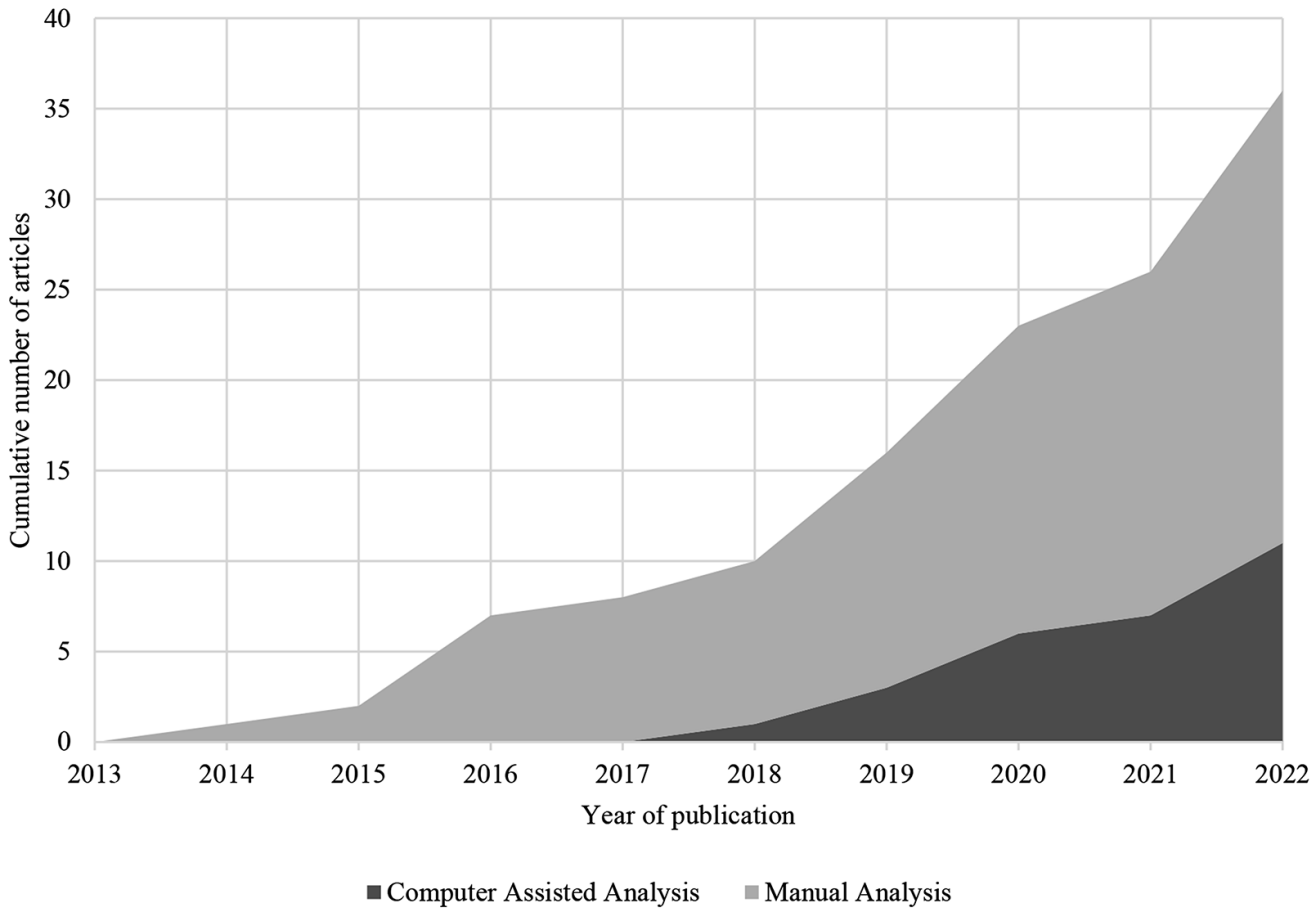
Cumulative number of articles published each year and their primary method of analysis

A total of 36 articles met all inclusion criteria and were synthesised in the results. The search yielded 10 articles (27.8%) which were published in 2022, the year the search was conducted. A further 15 articles were published within the previous three years from 2019 to 2021 (41.7%) and 11 were published in 2018 or earlier (30.6%). Figure 2 illustrates the growth in the cumulative number of peer-reviewed publications each year.

### Social media platforms and unit of analysis

As shown in Table 1, various social media platforms were used for the collection of data. Of the 36 included studies, the majority (*n* = 22, 61.1%) analysed data collected from Twitter. This was followed by 5 studies analysing Sina Weibo (13.9%), 4 studies analysing YouTube (11.1%), 2 studies analysing Instagram (5.6%), 1 study analysing TikTok (2.7%) and 1 study analysing Pinterest (2.7%). One study collected data from a variety of social media platforms (2.7%).

The unit/s of analysis (element of social media post analysed) also varied between studies (Refer to Table 2). A total of 28 (77.8%), primarily comprising the Twitter and Sina Weibo studies, analysed text-based data. Three studies analysed images (8.3%), two of which also involved analysis of associated captions (5.6%). Four studies analysed video-based content (11.1%). In total 8 studies (22.2%) conducted an analysis of comments associated with social media posts and 15 (41.7%) analysed reach metrics such as post likes and shares. Only 3 studies (8.3%) included an analysis of any content linked in a social media post such as an external website, and 14 (38.9%) collected and analysed data based on the social media profile type or demographics of content posters.

**Table 1:** Key characteristics of included studies (*n*=36)

Characteristic	N (%)	Study citation/s
Social media data source		
Twitter	22 (61.6%)	[20, 24–43]
Sina Weibo	5 (13.9%)	[44–48]
YouTube	4 (11.1%)	[49–52]
Instagram	2 (5.6%)	[53, 54]
TikTok	1 (2.8%)	[55]
Pinterest	1 (2.8%)	[56]
Other	1 (2.8%)	[57]
Mental Health Condition		
Schizophrenia/psychosis	14 (38.9%)	[19, 24, 27–32, 39, 40, 43, 45, 49, 57]
Mental health/mental illness	13 (36.1%)	[20, 25, 33, 35–38, 41, 42, 52–55]
Depression	12 (33.3%)	[26, 27, 29, 39, 40, 44–48, 50, 56]
Bipolar disorder	4 (11.1%)	[25, 27, 34, 56]
Other (Psychiatry terms, ASD, ED)	3 (8.3%)	[27, 29, 40]
Obsessive compulsive disorder	3 (8.3%)	[27, 29, 40]
Anxiety	2 (5.6%)	[27, 29]
Trichotillomania	1 (2.8%)	[51]
Social media language		
English	27 (75.0%)	[19, 20, 25, 26, 28–31, 33–43, 50–57]
Chinese	5 (13.9%)	[44–48]
Greek	2 (5.6%)	[24, 49]
Turkish	1 (2.8%)	[32]
French	1 (2.8%)	[27]
Finnish	1 (2.8%)	[49]
Year of Publication		
2022	10 (27.8%)	[27, 29, 30, 32, 38, 42, 51–53, 56]
2021	3 (8.3%)	[33, 48, 54]
2020	7 (19.4%)	[25, 28, 35–37, 45, 50]
2019	6 (16.7%)	[19, 20, 34, 40, 41, 43]
2018	2 (5.6%)	[44, 46]
2017	1 (2.8%)	[57]
2016	5 (13.9%)	[24, 26, 47, 49, 56]
2015	1 (2.8%)	[31]
2014	1 (2.8%)	[39]
Location of Researchers		
United States	19 (52.8%)	[20, 25, 26, 28, 31, 34–36, 41–43, 46–48, 50, 53–56]
Australia	1 (2.8%)	[39]
Canada	4 (11.1%)	[51, 52, 55, 57]
United Kingdom	5 (13.9%)	[19, 29, 30, 33, 40]
China	4 (11.1%)	[44–46, 48]
New Zealand	1 (2.78%)	[38]
Finland	2 (5.6%)	[24, 49]
Greece	2 (5.6%)	[24, 49]
Spain	1 (2.8%)	[43]
Turkey	1 (2.8%)	[32]
Israel	2 (5.6%)	[20, 25]
Netherlands	2 (5.6%)	[37, 38]

### Mental health condition/s in focus

The studies analysed social media content relating to one or more mental health conditions as per the review inclusion criteria (Refer to Table 1). The most frequent mental health condition was schizophrenia/psychosis, with content analysed in 14 studies (38.9%). This was closely followed by studies focused on mental health/mental illness content more broadly (*n* = 13, 36.1%), for example by searching for posts using #mentalhealth or ‘mental illness’, and studies which analysed depression (*n* = 12, 33.3%) Four included studies focused on bipolar disorder (11.1%), three studies focused on obsessive compulsive disorder (8.3%), only two focused on anxiety (5.6%) and one specifically focused on trichotillomania (2.8%).

Although the majority of studies focus solely on social media content related to one mental health condition, four studies (11.1%) include multiple health conditions and compare analysis results between each condition. Budenz et al. [25] compares content related to mental health/mental illness to content specific to bipolar disorder, while Jansli et al. [29] compares seven different mental health conditions. Both Li et al. [45] and Reavley and Pilkington [39] offer a comparison of schizophrenia/psychosis and depression related social media content. Four studies also incorporated a comparison between mental and physical health conditions into research aims. Studies compare mental ill-health content to diabetes [24, 31, 40, 43], cancer [40, 43], Alzheimer’s disease [43], HIV/AIDS [40, 43], asthma [40] and epilepsy [40].

### Social media content language and location of researchers

The inclusion criteria specified that studies must be published in English, but studies did not necessarily need to analyse English-based social media content. While 75.0% of studies did analyse English content (*n* = 27), five studies analysed Chinese content (13.9%), two studies analysed Greek content (5.6%), and Turkish, French, and Finnish social media content were each analysed in one study (8.4%) (Refer to Table 1).

Over half of the literature in this field is published by researchers affiliated with institutions within the United States (*n* = 19, 52.8%). This is followed by five studies from researchers in the United Kingdom (13.9%), four studies from China (11.1%), four studies from Canada (11.1%), and the remaining articles from researchers in Finland, Greece, Israel, Australia, New Zealand, Spain, Netherlands, and Turkey (*n* = 11, 30.6%).

### Study design

#### Data collection methods

The specific method of data collection varied based on the social media platform analysed. In most studies, authors applied a specific hashtag search relevant to the mental health topic of interest (e.g., #mentalhealth) or entered keywords into the social media platform search bar (e.g., “schizophrenia”). Given the volume of data posted to social media, most studies limited the collection of data to a specified time period, which ranged drastically between studies from 1 day to 10 years.

Several studies aimed to analyse mental health-related social media content based on a particular event or public health campaign, which dictated the timeframe of data collection. Makita et al. [33] collected data and analysed discourse specifically during Mental Health Awareness Week and Saha et al. [41] collected data only on World Mental Health Awareness Day. A study by Budenz et al. [20] collected data before and after a mass shooting event in the United States to identify changes in mental illness stigma messaging. Two studies analysed social media responses to the mental ill-health disclosure of professional athletes [36, 54], and one study collected data using the hashtag ‘#InHonorofCarrie’ to examine mental health-related content after the death of mental health advocate and actress Carrie Fisher [35].

While some authors analysed all posts identified in their social media search, others used specific inclusion/exclusion criteria and/or selection methods to limit the number of posts for further analysis. These included random selection of posts in the search result, selecting only every ‘x’th post, selecting the most viewed/liked/commented posts and/or selecting the first ‘x’ number of posts appearing in search results or each page of search results.

### Primary data analysis methods

While all included studies involved analysis of data extracted from social media, the method of analysis differed between studies (Refer to Table 2). The majority of studies conducted analysis through manual human-based coding (*n* = 25, 69.4%), of which 24 utilised some form of content analysis (*n* = 24, 66.7%). A total of eight (22.2%) content analysis studies employed an inductive coding approach in which themes were generated from the ‘ground up’ based on the data, while nine studies (25%) employed a deductive approach in which a coding framework was developed prior to the commencement of coding based on previous research and/or author expertise. However, six studies (16.7%) used a combination of approaches, in which a codebook was initially developed, but was inductively refined through a preliminary coding process. Only one study performed an inductive thematic analysis of social media content (2.8%), and one study used a combination of deductive content analysis and inductive thematic analysis to answer research questions (2.8%).

**Table 2:** Data analysis features of included studies (*n*=36)

Methodological Feature	N (%)	Study citation/s
Manual Analysis		
Deductive content analysis	9 (25%)	[19, 24, 29, 39, 46, 47, 49, 50, 54]
Deductive/inductive content analysis	6 (16.7%)	[31, 35, 40, 43, 51, 55]
Inductive content analysis	8 (22.2%)	[26–28, 32, 33, 36, 56, 57]
Inductive thematic analysis	1 (2.8%)	[52]
Deductive content analysis & inductive thematic analysis	1 (2.8%)	[34]
Computer Assisted Analysis		
Manual content analysis and machine learning	5 (13.9%)	[20, 25, 30, 41, 53]
Linguistic analysis	3 (8.3%)	[44, 45, 48]
Sentiment analysis and topic modelling	2 (5.6%)	[37, 38]
N-gram language modelling	1 (2.8%)	[42]
Unit of Analysis		
Text	28 (77.8%)	[19, 20, 24–35, 37–48, 53, 57]
Image	3 (8.3%)	[33, 53, 56]
Caption	2 (5.6%)	[53, 56]
Comments	8 (22.2%)	[35, 36, 47, 52–56, 57]
Video	4 (11.1%)	[49–51, 55]
Likes, shares, etc.	15 (41.7%)	[20, 24, 25, 32, 34, 39, 41, 43, 47–50, 53, 55, 56]
Links	3 (8.3%)	[28, 33, 46]
Social media profile/demographics	14 (38.9%)	[19, 20, 24, 26, 28, 33, 39, 41, 44–47, 49, 51]
Co-occurring hashtags	1 (2.8%)	[41]
Follower count	1 (2.8%)	[26]

In total five studies (13.9%) used human-based coding in combination with computer-assisted coding, whereby an initial sample of human coded data was used to develop a machine learning model which could subsequently analyse a large volume of data. Aside from content analysis and thematic analysis, three studies conducted software-mediated linguistic analysis (8.3%) and two studies involved sentiment analysis and topic modelling (8.3%) and one used language modelling (2.5%). Figure 2 illustrates the cumulative number of articles published each year and the primary analysis employed. The figure demonstrates that an article utilising a computer-assisted approach was first published in 2018, and there has since been a surge in the number of studies adopting these tools for analysis.

### Coding frameworks

The authors who utilised a deductive approach to content analysis, either developed their own coding framework, or adopted a framework previously developed and reported in the literature. Frameworks varied greatly between studies but often included coding the type of social media profile (e.g., individual, consumer, health professional, organisation), the type of mental health-related content (e.g., personal experience, awareness promotion, advertising, news media, personal opinion/dyadic interaction) and/or the broader topic or context of posts (e.g., politics, everyday social chatter, culture/entertainment, mental health, news, awareness campaigns). Some studies also chose to categorise mental health-related content as either ‘medical’ (e.g., diagnosis, treatment, prognosis) or ‘non-medical’ before further classification.

In terms of coding for representation or attitudes towards mental ill-health, most studies coded for stigma, variously defined. In some studies, this was merely the presence or absence of stigma for each unit of analysis (e.g., was there stigmatising content in the tweet or not), but in others stigma was further broken down into more specific types of stigma. For example, the coding framework developed by Reavley and Pilkington [39] includes stigmatising attitude subthemes such as ‘social distance’, ‘dangerousness’, and ‘personal weakness’. In some studies, trivialisation has been classed as stigma, while in others a separate coding category has been created for any posts which are deemed to be trivialising, mocking or sarcastic towards mental ill-health. Another common approach in the included studies was to code for the valence or overall sentiment of each unit of analysis, in which categories included positive, neutral or negative polarity, or classified tone as positive or pejorative. Some authors analysed the use of mental health related terminology and categorised this based on whether terms are misused or employed metaphorically.

### Quality appraisal

The studies were appraised using the CASP tool for qualitative research, which does not calculate a final score or provide an overall grade of quality. A total of 37 studies met all the review inclusion criteria and were appraised by reviewers. A breakdown of appraisal results for each CASP item is presented in Additional File 2. The criteria in which the highest number of studies received a rating of ‘no’ related to the rigour of data analysis (*n* = 6, 16.7%) and clarity of stating findings (*n* = 6, 16.7%). Based on the results of the appraisal and after discussion between all authors, one study was excluded from the review synthesis due to lack of clarity in reporting methods [58].

## Discussion

This review summarised the current literature investigating the representation of mental ill-health on social media, in particular focussing on methodological design. While human-based content analysis was the dominant means of qualitative data analysis, a limited number of studies employed computer-based techniques. The results also indicated an uneven distribution in the social media platforms selected for data collection, as well as the unit/s of analysis. These findings suggest some important methodological gaps in the literature.

### A growing area of research interest

The results demonstrate that almost 70% of all studies in this field were published within the last four years, from 2019 to 2022, suggesting this is an emerging area of interest in the academic literature. Social media research has been used to identify the attitudes and opinions of the public regarding many topics, but appears to have rapidly gained favour amongst researchers during the COVID-19 pandemic, researching public perceptions of issues such as vaccination [59], healthcare staff [60], restrictions [61] and the pandemic more broadly [62–64]. Perhaps the surge in publications relating to the representation of mental ill-health on social media is reflective of a wider trend towards this type of research and an acknowledgement amongst researchers of the power of social media data. Social media presents real-time data to capture current public perceptions about a topic and the opportunity to monitor changes over time [62]. However, it must also be acknowledged that the recent growth in publications found may also be reflective of a societal shift towards increased acceptance of using online social media as an appropriate forum for mental health-related discourse, triggering subsequent research interest [65, 66].

### The dominance of Twitter-based research

Our review revealed an uneven distribution of social media platforms studied within the current literature. Over 50% of the included studies collected data from the text-based social media platform Twitter and a further five studies analysed Sina Weibo data, a Chinese microblogging site highly reminiscent of Twitter. These results align with the findings from other systematic reviews into social media-based research, which demonstrate a skewed focus towards text-based data sources [67, 68]. This dominance in the research landscape is likely due to methodological considerations. Twitter is an open-source platform and users can choose not to reveal their identity in profile ‘handles’. The text-based nature of the data also ensures analysis is relatively easier and permits the use of machine learning approaches.

Unfortunately, the emphasis on Twitter limits the scope of this body of research and does not accurately reflect the relative popularity of social media platforms. As of 2023, Facebook has the highest number of global monthly active users (MAUs) at more than 2.9 billion, yet none of the included studies in this review collected data from this platform. This is likely because collecting data on Facebook and other direct messaging platforms without breaching the privacy of users remains an ethical challenge [67]. Image and video-sharing platforms have seen rapid growth in popularity in the last few years, yet only represent a minority of the studies in this review. Instagram has over 2 billion MAUs, and the video-based platform TikTok has over 1 billion, suggesting a much higher share of the social media market than Twitter at 556 million and Sina Weibo at 584 million MAUs [69].

Such dominance in the use of Twitter means that certain populations and age groups are underrepresented in the current research. Twitter is known to have an older demographic of users, with 38.5% aged 25–34 years and 20.7% aged 35–49 years [70]. By comparison, TikTok has become a popular platform for teenagers and young adults, with 67.3% aged under 24 years and only 5.97% aged 35–44 years [71]. Young people are known to experience a higher rate of mental illness in comparison to older age groups, but their engagement with mental health care is often poor causing a delay in help-seeking behaviour [4, 9, 72]. Thus, future social media-based research into the representation of mental health conditions on platforms predominantly frequented by younger users has the potential to add significant value to this body of literature.

### Analysis of social media content

While the majority of included studies employed content analysis (*n* = 24, 66.7%), their processes varied considerably. It is worth noting that nine of these 24 studies, followed a deductive dominant approach to coding, while a further seven included a deductive element. A deductive (or sometimes termed ‘directive’) approach to content analysis is most appropriate where existing research findings, conceptual frameworks or theories can be used to guide codebook development [73, 74]. Thus, given that there is extensive previous literature related to the representation of mental ill-health (albeit not necessarily in social media), and in particular frameworks for mental illness stigma, it is appropriate to take a deductive approach [75, 76]. However, introducing an inductive element to the approach, in which the initial codebook is inductively refined through initial coding stages can result in coding categories more suited to the specific social media data extracted from the platform of interest and potentially provide more nuanced analysis [77].

It should also be noted the apparent dearth of studies in this field adopting thematic analysis. There are several reasons why this may be the case, the foremost being the volume of data for analysis on social media. It is widely held in the literature that the choice of content analysis versus thematic analysis is a question of wide application versus deep analysis [78]. Due its alignment with quantitative research, content analysis can be more suited to larger data sets, whereas thematic analysis allows for greater immersion in the data and depth of understanding [78]. While both are of value, in the case of social media data where researchers are aiming to understand public representations and attitudes towards mental illness, content analysis can provide the wider analysis required for research questions.

Review of the current literature also suggested the coding frameworks adopted by the included studies vary greatly, making comparison of their findings challenging. Each study defined the concept of stigma differently through their approach to coding, for example both Jansli et al. [29] and Jilka et al. [30] simply identified whether content was stigmatising or not. Conversely, Budenz et al. [25] coded for the presence or absence of mental illness stigma and then specifically coded for violence-related mental illness stigma as the study aimed to identify changes in tweet content before and after a mass shooting event. Meanwhile, Reavley and Pilkington [39] took the coding process one step further and developed a detailed coding framework which groups different types of stigmatising attitudes such as ‘beliefs that mental illness is due to personal weakness’, ‘people with mental illness are dangerous’ and ‘desire for social distance from the person’. In critically analysing the methodological approaches of these studies, it must be acknowledged that stigma is a broad concept containing many nuances. In order to gain a deep understanding of societal perceptions and attitudes towards mental ill-health, coding frameworks should be developed with these nuances in mind and reflect the many aspects of stigmatising attitudes. Content analysis should avoid a ‘tick box’ approach to the identification of stigma, and instead aim for a richer understanding of mental ill-health perceptions.

Of the studies which employed content analysis, the vast majority used a manual approach in which human researchers hand coded the data. However, more recently machine learning techniques have been applied to the field. For example, Saha et al. [41] hand coded a sample of 700 tweets and used these to develop a machine learning framework to automatically infer the topic of the remaining 13,517 tweets. Several studies also used specialised packages such as Linguistic Inquiry and Word Count software to extract the psycholinguistic features from social media data and obtain quantitative counts [37, 44, 45]. The clear advantage of these computerised methods is that they allow researchers to analyse much larger volumes of data and reduce the manual labour and time involved in the analysis process. While these studies undoubtedly add value to the body of literature, there still remains a place for the process of manual human coding, especially in the case of more detailed coding frameworks, which can offer more nuanced insights. Although technology is rapidly advancing, manual human coding also remains the only viable means of analysis for researchers intending to interpret image and video-based data.

### Quality of studies and frequent issues

Critical appraisal of the included studies was conducted using the CASP tool for qualitative research [79]. As was described in the methods, this was deemed the most appropriate tool for the appraisal, yet authors still needed to modify and adapt the tool for the purposes of this review. Given the difficulty in finding an appropriate critical appraisal tool for studies which involve analysis of social media-based content and the apparent growth in researcher interest for this study design, the authors advocate for the need of the development of a more specific appraisal tool.

The authors noted a few frequent issues which lowered the quality of included studies and should be addressed in future research in the field. Firstly, multiple studies did not describe the process of codebook development with transparency and if the approach was deductive did not indicate the previous literature which assisted this process. The coding framework is key to ensuring rigorous data analysis and generating meaningful findings, and its development should therefore be described in sufficient detail. The reviewers also noted inconsistency in study coding protocols for content analysis studies. In this type of analysis, reliability is of paramount importance, and previous methodological literature highlights the need to establish intercoder reliability (ICR) [80, 81]. At least two coders are needed to independently analyse data [81], or alternatively two coders can analyse a sample of data and if sufficient intercoder reliability is achieved, one coder can complete the remaining analysis [82]. Yet, some studies utilised only a single coder, did not establish or report measures of intercoder reliability, or were unclear in their reporting of the coding protocol. Content analysis is susceptible to human biases during the coding process, and thus it is essential to minimise these risks through a robust protocol.

### Limitations of social media-based research

Although the strengths of social media-based research are numerous, there are several key limitations to this type of research. Many studies utilise ‘hashtags’ to search and identify content relevant to their topic of interest. However, not everyone who posts on social media uses hashtags, and these are often employed as a means to generate followers [83]. There are also some technical challenges in the data collection process whereby researchers must use external programs such as a Twitter Application Programming Interface to search for data which only permits access to a portion of all tweets.

Another important consideration is that findings cannot necessarily be generalised to the wider community. Although social media is a significant aspect of life for many, some demographics use and post on social media more frequently than others, for example women and younger age groups [84, 85]. Not everyone uses and interacts with social media in the same way, so this type of research cannot be used to interpret the opinions and perspectives of the broader population.

Social media-based research is also somewhat constrained by ethical concerns regarding user privacy. Studies are often limited to the use of data extracted from public profiles, which in turn may bias the type of data collected. Mental health is an inherently sensitive topic, and thus analysis of mental health content posted to private social media profiles may yield additional insights.

### Limitations of the review

This systematic review is subject to several limitations which must be noted. Firstly, the scope of this review was limited to identification and analysis of the methods used in the included studies and did not extend to synthesis of results. Future review articles may wish to focus on synthesis of results, although their highly heterogenous nature is likely to prevent meta-analysis. Secondly, the search was filtered to include only articles which were published in the English language. This may have missed relevant studies published in a language other than English, although the review did include several studies focused on social media content posted in Chinese, Greek, Turkish, French, and Finnish. The database searches were also limited to peer-reviewed publications as per convention for systematic literature reviews, however this search approach could potentially miss peer-reviewed conference proceedings and industry reports [67].

## Conclusion

This review is the first to systematically identify, summarise and critically evaluate the available literature focused on the representation of mental ill-health on social media. The review analysed current methodologies employed by these studies and critically evaluated strengths and weaknesses of the various approaches adopted by researchers. The results highlight the need to shift away from text-based social media research such as Twitter, towards the more popular and emerging image and video-based platforms. The utility of both manual and computer-assisted content analysis was discussed, and reviewers concluded that both make valuable contributions to the body of research. Future research could aim to investigate how social media representation of mental illness translates to ‘real-life’ attitudes and instances of stigmatising behaviour, as well as the help-seeking behaviours of those experiencing symptoms of mental ill-health. Along with many other non-communicable chronic diseases, the rate of mental illness continues to grow, presenting an urgent public health challenge. This field of research can help to develop a deeper understanding of societal attitudes towards mental ill-health and reveal the information those suffering from mental ill-health are exposed to on social media. Through this knowledge, mental and public health professionals can create more targeted and effective campaigns to combat negative representations of mental ill-health using social media as a medium.

### Electronic supplementary material

Below is the link to the electronic supplementary material.

Supplementary Material 1

Supplementary Material 2

## Data Availability

The datasets used and/or analysed during the current study are available from the corresponding author on reasonable request.

## Abbreviations

DALY: Disability-adjusted life year
COVID-19: Coronavirus disease 2019
PROSPERO: International Prospective Register of Systematic Reviews
PRISMA: Preferred Reporting Items for Systematic reviews and Meta-Analyses
DSM-V: Diagnostic and Statistical Manual of Mental Disorders, Fifth Edition
CASP: Critical Appraisal Skills Programme
ASD: Autism spectrum disorder
ED: Eating disorder
HIV/AIDs: Human immunodeficiency virus/Acquired immunodeficiency syndrome
MAU: Monthly active users
