# Supplementary material for: What methods are used to examine representation of mental ill-health on social media? A systematic review
Source: BMC Psychol. 2024 Feb 29;12:105. doi: 10.1186/s40359-024-01603-1 (PMC10905888; doi:10.1186/s40359-024-01603-1)
Supplement: Supplementary file 1 — Supplementary Material 1 [file 40359_2024_1603_MOESM1_ESM.docx]

# Additional File 1: Search strategy

MEDLINE (via Ovid)

1. Exp Social Media/
2. ("social media" or "social platform" or "social networking site*" or "online social network*" or user-generated or "user generated" or "online activit*" or blog or "web log" or "online forum" or "discussion board" or "message board" or Instagram or Facebook or TikTok or Twitter or Tweet* or Pinterest or Snapchat or Tumblr or Reddit or YouTube or MySpace or Flickr or Whatsapp or BeReal or Weibo or Baidu or WeChat or Qzone or QQ).ti,ab.
3. 1 OR 2
4. Exp mental health/ or exp anxiety disorders/ or exp "bipolar and related disorders"/ or exp mood disorders/ or "schizophrenia spectrum and other psychotic disorders"/ or exp schizophrenia/
5. ((Mental adj3 (health or disorder* or illness* or condition)) or ("Schizo*" or Psychot* or Bipolar or depress* or anxiety or panic or GAD or obsessive-compulsive or "obsessive compulsive" or OCD)).ti,ab.
6. 4 OR 5
7. Exp qualitative research/
8. (((Content or discourse or thematic or linguistic or sentiment or text or qualitative) adj3 analy*) or "qualitative research" or fram* or "text min*").ti,ab.
9. (Represent* or misrepresent* or misuse or define* or views* or portray* or perception* or perceive* or attitude* or Stigma* or self-stigma* or "self stigma*" or discrim* or prejudic* or "internal stigma*" or trivial* or romant* or glamo*).ti,ab.
10. 7 OR 8 OR 9
11. 3 AND 6 AND 10

1,541 results

Filter by Journal Article [Publication Type] = 1520

PsycINFO (via Ovid)

1. Exp Social Media/
2. ("social media" or "social platform" or "social networking site*" or "online social network*" or user-generated or "user generated" or "online activit*" or blog or "web log" or "online forum" or "discussion board" or "message board" or Instagram or Facebook or TikTok or Twitter or Tweet* or Pinterest or Snapchat or Tumblr or Reddit or YouTube or MySpace or Flickr or Whatsapp or BeReal or Weibo or Baidu or WeChat or Qzone or QQ).ti,ab.
3. 1 OR 2
4. Exp mental health/ or exp anxiety disorders/ or exp "bipolar and related disorders"/ or exp mood disorders/ or "schizophrenia spectrum and other psychotic disorders"/ or exp schizophrenia/
5. ((Mental adj3 (health or disorder* or illness* or condition)) or ("Schizo*" or Psychot* or Bipolar or depress* or anxiety or panic or GAD or obsessive-compulsive or "obsessive compulsive" or OCD)).ti,ab.
6. 4 OR 5
7. Exp qualitative research/
8. (((Content or discourse or thematic or linguistic or sentiment or text or qualitative) adj3 analy*) or "qualitative research" or fram* or "text min*").ti,ab.
9. (Represent* or misrepresent* or misuse or define* or views* or portray* or perception* or perceive* or attitude* or Stigma* or self-stigma* or "self stigma*" or discrim* or prejudic* or "internal stigma*" or trivial* or romant* or glamo*).ti,ab.
10. 7 OR 8 OR 9
11. 3 AND 6 AND 10

1,685 results

Filter by Peer Reviewed Journal [Publication Type] = 1220

CINAHL (via EBSCO)

Social Media = S1

1. (MH "Social Media+") OR (MH "Blogs") OR (MH "Online Social Networking")
2. ("social media" or "social platform" or "social networking site*" or "online social network*" or user-generated or "user generated" or "online activit*" or blog or "web log" or "online forum" or "discussion board" or "message board" or Instagram or Facebook or TikTok or Twitter or Tweet* or Pinterest or Snapchat or Tumblr or Reddit or YouTube or MySpace or Flickr or Whatsapp or BeReal or Weibo or Baidu or WeChat or Qzone or QQ) 🡪 Select TI (Title)
3. ("social media" or "social platform" or "social networking site*" or "online social network*" or user-generated or "user generated" or "online activit*" or blog or "web log" or "online forum" or "discussion board" or "message board" or Instagram or Facebook or TikTok or Twitter or Tweet* or Pinterest or Snapchat or Tumblr or Reddit or YouTube or MySpace or Flickr or Whatsapp or BeReal or Weibo or Baidu or WeChat or Qzone or QQ) 🡪 Select AB (Abstract)

Mental Health = S2

1. (MH "Mental Health") OR (MH "Anxiety Disorders") OR (MH "Bipolar Disorder+") OR (MH "Depression+") OR (MH "Schizophrenia+") OR (MH "Obsessive-Compulsive Disorder+")
2. ((Mental N3 (health or disorder* or illness* or condition)) or ("Schizo*" or Psychot* or Bipolar or depress* or anxiety or panic or GAD or obsessive-compulsive or "obsessive compulsive" or OCD)) 🡪 Select T1 (Title)
3. ((Mental N3 (health or disorder* or illness* or condition)) or ("Schizo*" or Psychot* or Bipolar or depress* or anxiety or panic or GAD or obsessive-compulsive or "obsessive compulsive" or OCD)) 🡪 Select AB (Abstract)

Methodology & Specific Terms = S3

1. (MH "Qualitative Studies")
2. (((Content or discourse or thematic or linguistic or sentiment or text or qualitative) N3 analy*) or "qualitative research" or fram* or "text min*") 🡪 Select TI (Title)
3. (((Content or discourse or thematic or linguistic or sentiment or text or qualitative) N3 analy*) or "qualitative research" or fram* or "text min*") 🡪 Select AB (Abstract)
4. (Represent* or misrepresent* or misuse or define* or views* or portray* or perception* or perceive* or attitude* or stigma* or self-stigma* or "self stigma*" or discrim* or prejudic* or "internal stigma*" or trivial* or romant* or glamo*) 🡪 Select TI (Title)
5. (Represent* or misrepresent* or misuse or define* or views* or portray* or perception* or perceive* or attitude* or stigma* or self-stigma* or "self stigma*" or discrim* or prejudic* or "internal stigma*" or trivial* or romant* or glamo*) 🡪 Select AB (Abstract)

Combined Search = S4

1. S1 AND S2 AND S3

1,340 results

Filter by Academic Journals [Source Types], English [Language] = 1,260

PROQUEST Public Health Database, Psychology Database and Computer Science Database

Social Media = S1

1. mesh.Exact("Social Media")
2. ("social media" or "social platform" or "social networking site*" or "online social network*" or user-generated or "user generated" or "online activit*" or blog or "web log" or "online forum" or "discussion board" or "message board" or Instagram or Facebook or TikTok or Twitter or Tweet* or Pinterest or Snapchat or Tumblr or Reddit or YouTube or MySpace or Flickr or Whatsapp or BeReal or Weibo or Baidu or WeChat or Qzone or QQ) 🡪 Document title -- TI
3. ("social media" or "social platform" or "social networking site*" or "online social network*" or user-generated or "user generated" or "online activit*" or blog or "web log" or "online forum" or "discussion board" or "message board" or Instagram or Facebook or TikTok or Twitter or Tweet* or Pinterest or Snapchat or Tumblr or Reddit or YouTube or MySpace or Flickr or Whatsapp or BeReal or Weibo or Baidu or WeChat or Qzone or QQ) 🡪 Abstract – AB

Mental Health = S2

1. mesh.Exact("Anxiety Disorders" OR "Bipolar Disorder" OR "Obsessive-Compulsive Disorder" OR "Schizophrenia" OR "Depression" OR "Mental Health" OR "Anxiety")
2. ((Mental Near/3 (health or disorder* or illness* or condition)) or ("Schizo*" or Psychot* or Bipolar or depress* or anxiety or panic or GAD or obsessive-compulsive or "obsessive compulsive" or OCD)) 🡪 Document title -- TI
3. ((Mental Near/3 (health or disorder* or illness* or condition)) or ("Schizo*" or Psychot* or Bipolar or depress* or anxiety or panic or GAD or obsessive-compulsive or "obsessive compulsive" or OCD)) 🡪 Abstract – AB

Methodology & Specific Terms = S3

1. mesh.Exact("Qualitative Research")
2. ((Content OR discourse OR thematic OR linguistic OR sentiment OR text OR qualitative) Near/3 analy*) OR "qualitative research" OR fram* OR "text min*" 🡪 Document title – TI
3. ((Content OR discourse OR thematic OR linguistic OR sentiment OR text OR qualitative) Near/3 analy*) OR "qualitative research" OR fram* OR "text min*" 🡪 Abstract – AB
4. (Represent* or misrepresent* or misuse or define* or view* or portray* or perception* or perceive* or attitude* or Stigma* or self-stigma* or "self stigma*" or discrim* or prejudic* or "internal stigma*" or trivial* or romant* or glamo*) 🡪 Document title – TI
5. (Represent* or misrepresent* or misuse or define* or view* or portray* or perception* or perceive* or attitude* or Stigma* or self-stigma* or "self stigma*" or discrim* or prejudic* or "internal stigma*" or trivial* or romant* or glamo*) 🡪 Abstract – AB

Combined Search

S1 AND S2 AND S3

1,680 results

Filtered by Scholarly Journals [Source type], English [Language] = 1,172

SCOPUS

1. ("social media" or "social platform" or "social networking site*" or "online social network*" or user-generated or "user generated" or "online activit*" or blog or "web log" or "online forum" or "discussion board" or "message board" or Instagram or Facebook or TikTok or Twitter or Tweet* or Pinterest or Snapchat or Tumblr or Reddit or YouTube or MySpace or Flickr or Whatsapp or BeReal or Weibo or Baidu or WeChat or Qzone or QQ)

AND

1. ((Mental W/3 (health or disorder* or illness* or condition)) or ("Schizo*" or Psychot* or Bipolar or depress* or anxiety or panic or GAD or obsessive-compulsive or "obsessive compulsive" or OCD))

AND

1. ( ((Content OR discourse OR thematic OR linguistic OR sentiment OR text OR qualitative) W/3 analy*) OR "qualitative research" OR fram* OR "text min*" ) OR (Represent* or misrepresent* or misuse or define* or view* or portray* or perception* or perceive* or attitude* or Stigma* or self-stigma* or "self stigma*" or discrim* or prejudic* or "internal stigma*" or trivial* or romant* or glamo*)

5,999 results

Limited to Article [Document Type], English [Language] = 4,349

Search within ‘Article title, Abstract, Keywords’
