# Supplementary material for: What methods are used to examine representation of mental ill-health on social media? A systematic review
Source: BMC Psychol. 2024 Feb 29;12:105. doi: 10.1186/s40359-024-01603-1 (PMC10905888; doi:10.1186/s40359-024-01603-1)
Supplement: Supplementary file 2 — Supplementary Material 2 [file 40359_2024_1603_MOESM2_ESM.docx]

**Additional File 2**

**Table S1.** CASP critical appraisal of included studies.

| Citation | 1. Was there a clear statement of the aims of the research? | 2. Is a qualitative methodology appropriate? | 3. Was the research design appropriate to address the aims of the research? | 5. Was the data collected in a way that addressed the research issue? | 7. Have ethical issues been taken into consideration | 8. Was the data analysis sufficiently rigorous? | 9. Is there a clear statement of findings? | 10. How valuable is the research? |
| --- | --- | --- | --- | --- | --- | --- | --- | --- |
| (Alvarez-Mon et al., 2019) | Yes | Yes | Yes | Yes | Yes | Can’t tell | Yes | Valuable |
| (Athanasopoulou & Sakellari, 2016) | Yes | Yes | Yes | Yes | Can’t tell | Can’t tell | Yes | Valuable |
| (Athanasopoulou et al., 2016) | Yes | Yes | Yes | Yes | Yes | No | Yes | Valuable |
| (Basch et al., 2022) | Yes | Yes | Can’t tell | Can’t tell | Yes | No | Yes | Valuable |
| (Budenz et al., 2020) | Yes | Yes | Yes | Can’t tell | Yes | Yes | Yes | Valuable |
| (Budenz et al., 2019) | Yes | Yes | Yes | Yes | Yes | No | Yes | Valuable |
| (Cavazos-Rehg et al., 2016) | Yes | Yes | Yes | Yes | Yes | Yes | Can’t tell | Valuable |
| (Delanys et al., 2022) | Yes | Yes | Yes | Can’t tell | Yes | Yes | No | Valuable |
| (Devendorf et al., 2020) | Can’t tell | Yes | Yes | Can’t tell | Can’t tell | Can’t tell | Yes | Valuable |
| (Ghate et al., 2022) | Yes | Yes | Yes | Yes | Yes | Yes | Yes | Valuable |
| (Guidry et al., 2016) | Yes | Yes | Can’t tell | No | Can’t tell | Can’t tell | Can’t tell | Valuable |
| (Hernandez et al., 2020) | Yes | Yes | Yes | Can’t tell | Can’t tell | Can’t tell | Yes | Valuable |
| (Jansli et al., 2022) | Yes | Yes | Yes | Yes | Yes | Yes | Yes | Valuable |
| (Jilka et al., 2022) | Yes | Yes | Yes | Can’t tell | Yes | Can’t tell | Yes | Valuable |
| (Joseph et al., 2015) | Yes | Yes | Yes | Yes | Yes | Can’t tell | No | Valuable |
| (Kara & Şenel Kara, 2022) | Yes | Yes | Yes | Yes | Can’t tell | Can’t tell | Yes | Valuable |
| (Ang Li et al., 2020) | Yes | Yes | Yes | No | Yes | Yes | No | Valuable |
| (A. Li et al., 2018) | Yes | Yes | Yes | No | Yes | No | Yes | Valuable |
| (Makita et al., 2021) | Yes | Yes | Yes | Yes | Yes | Yes | Yes | Valuable |
| (McLellan et al., 2022) | Yes | Yes | Yes | No | Yes | Yes | Yes | Valuable |
| (Nelson, 2019) | Yes | Yes | Can’t tell | Can’t tell | Yes | Can’t tell | Yes | Valuable |
| (Pan et al., 2018) | Yes | Yes | Yes | Yes | Yes | Yes | Yes | Valuable |
| (Park & Hoffner, 2020) | Yes | Yes | Yes | Yes | Can’t tell | Can’t tell | Yes | Valuable |
| (Parrott et al., 2020) | Yes | Yes | Yes | Yes | Can’t tell | Yes | Yes | Valuable |
| (Passerello et al., 2019) | Yes | Yes | Yes | Can’t tell | Yes | Yes | Yes | Valuable |
| (Pavelko & Wang, 2021) | Yes | Yes | Yes | Yes | Can’t tell | Can’t tell | No | Valuable |
| (Pavlova & Berkers, 2022) | Yes | Yes | Yes | Yes | Yes | Yes | Yes | Valuable |
| (Pavlova & Berkers, 2020) | Yes | Yes | Yes | Yes | Yes | Can’t tell | Yes | Valuable |
| (Reavley & Pilkington, 2014) | Yes | Yes | Yes | Yes | Yes | Yes | Yes | Valuable |
| (Robinson et al., 2019) | Yes | Yes | Yes | Yes | Yes | Can’t tell | Yes | Valuable |
| (Saha et al., 2019) | Yes | Yes | Yes | Can’t tell | Yes | Yes | No | Valuable |
| (Shigeta et al., 2017) | Yes | Yes | Yes | Can’t tell | Can’t tell | No | Yes | Valuable |
| (Stupinski et al., 2022) | Yes | Yes | Yes | Can’t tell | Can’t tell | Yes | Yes | Valuable |
| (Vidamaly & Lee, 2021) | No | Can’t tell | Can’t tell | No | Yes | No | Can’t tell | Not valuable |
| (W. Wang & Liu, 2016) | Yes | Yes | Yes | Yes | Can’t tell | Can’t tell | Yes | Valuable |
| (Wu & Hong, 2022) | Yes | Yes | Yes | Can’t tell | Can’t tell | Yes | Yes | Valuable |
| (Yu et al., 2021) | Yes | Yes | Yes | Yes | Yes | Yes | No | Valuable |
